# A New Multicolor Bioluminescence Imaging Platform to Investigate NF-κB Activity and Apoptosis in Human Breast Cancer Cells

**DOI:** 10.1371/journal.pone.0085550

**Published:** 2014-01-17

**Authors:** Laura Mezzanotte, Na An, Isabel M. Mol, Clemens W. G. M. Löwik, Eric L. Kaijzel

**Affiliations:** 1 Department of Radiology, Leiden University Medical Center, Leiden, The Netherlands; 2 Department of Endocrinology, Leiden University Medical Center, Leiden, The Netherlands; National Institutes of Health, United States of America

## Abstract

**Background:**

Evaluation of novel drugs for clinical development depends on screening technologies and informative preclinical models. Here we developed a multicolor bioluminescent imaging platform to simultaneously investigate transcription factor NF-κB signaling and apoptosis.

**Methods:**

The human breast cancer cell line (MDA-MB-231) was genetically modified to express green, red and blue light emitting luciferases to monitor cell number and viability, NF-κB promoter activity and to perform specific cell sorting and detection, respectively. The pro-luciferin substrate Z-DEVD-animoluciferin was employed to determine apoptotic caspase 3/7 activity. We used the cell line for the *in vitro* evaluation of natural compounds and *in vivo* optical imaging of tumor necrosis factor TNFα-induced NF-κB activation.

**Results:**

Celastrol, resveratrol, sulphoraphane and curcumin inhibited the NF-κB promoter activity significantly and in a dose dependent manner. All compounds except resveratrol induced caspase 3/7 dependent apoptosis. Multicolor bioluminescence *in vivo* imaging allowed the investigation of tumor growth and NF-κB induction in a mouse model of breast cancer.

**Conclusion:**

Our new method provides an imaging platform for the identification, validation, screening and optimization of compounds acting on NF-κB signaling and apoptosis both *in vitro* and *in vivo*.

## Introduction

In the last decades bioluminescent reporters have been employed extensively for the development of cell-based assays and *in vivo* imaging [1]–[5]. Molecular aspects of color variation in luciferase emission have been the subject of numerous studies: various mechanisms have been proposed [6] and multicolor reporter proteins have been created and applied in cell-based assays [7]–[10].

Multicolor bioluminescence systems present high emission quantum yields that confer high detectability in *in vitro* but also in *in vivo* settings. In particular, the combined use of D-luciferin-dependent luciferases with different peak emission wavelengths (‘multicolored’ luciferases)[11], with luciferases using different substrates like coelenterazine or vargulin [12]–[14] expanded the potential of cell-based assays and *in vivo* imaging. These multiplexed analyses scale down assay formats and enable the reduction and refinement of animal experimentation [15], [16].

New imaging tools will help in understanding the molecular mechanisms that lead to cancer progression and metastasis as well as resistance to chemotherapy. The development of such preclinical research tools, that ensures a faster and more accurate analysis of molecular pathways, is essential to improve the design and screening of new drugs and the diagnosis and treatment of cancer.

The Nuclear Factor-kappa B (NF-κB) signal transduction pathway has been identified as a key pathway in inflammation associated cancer, in cell transformation and tumor growth and in cell invasion and metastasis, especially in breast cancer [17], [18]. Understanding the mechanisms of NF-κB activation in tumor cells will facilitate development of means for cancer prevention and therapy [19]–[21]. Targeting the NF-κB activation pathway, commonly activated in breast cancer cells, is expected to lower the survival threshold even if NF-κB inhibition is generally insufficient for inducing pronounced apoptosis in cancer cells. NF-κB inhibitors can be used as adjuvants along with chemo -and radiotherapy or for cancer prevention. Different natural compounds have been discovered that directly or indirectly suppress NF-κB activity at key points along the activation pathway and they have been examined for chemoprevention, chemosensitization or adjuvants [22]–[26].

Effects of new candidate anticancer drugs on NF-κB signaling have been extensively investigated by classic cell-based approaches like transient transfection assays using NF-κB promoter-reporter plasmids [27] and electrophoresis mobility shift assays [28]. Optical tools and transgenic bioluminescent animals have been introduced to study the effects on NF-κB signaling, like p65-GFP fusions [29], IκBα-luciferase (IκBα-FLuc) fusion reporters [30] and NF-κB luc reporter mice [31].

In this study we developed and validated a new triple color cancer cell system generated by lentiviral transduction of the human breast cancer cell line MDA-MB-231 with different bioluminescent reporters. In particular, the click beetle green luciferase (CBG99) was used to monitor cell vitality while the red mutant of firefly luciferase (PpyRE9) monitored NF-κB promoter activity. The blue extGluc, a transmembrane form of Gaussia luciferase, served as a reporter for *in vitro* cell sorting and *ex vivo* cell analysis. Additionally, the use of the luciferase pro-substrate Z-DEVD-aminoluciferin, containing the DEVD tetrapeptide sequence recognized by caspase-3 and -7, allows the non-invasive imaging of apoptosis in luciferase expressing cells both *in vitro* and *in vivo* [32]. Upon activation of caspase-3 or -7 in apoptotic cells, the DEVD peptide is cleaved, and the liberated aminoluciferin reacts with luciferase to generate measurable light.

By means of this new imaging platform we investigated the effects of plant-derived natural compounds like celastrol, resveratrol, curcumin, betulinic acid and sulphoraphane on TNFα-induced NF-κB activation and apoptosis in human breast cancer cells. For the first time, analysis of NF-κB activation and tumor progression from cells to animal models using multicolored reporter genes for drug testing is reported.

## Experimental Procedures

### Ethical statement

Animal experiments were reviewed and approved by the Bioethics Committee of Leiden University, The Netherlands. All animals received human care in compliance with the “Code of Practice Use of Laboratory Animals in Cancer Research” (Inspectie W&V, July 1999).

### Cell culture and reagents

MDA-MB-231 human breast cancer cells (ATCC number HTB-26™) were maintained in Dulbecco's modified Eagle's medium (Invitrogen, Grand Island, U.S.A.) supplemented with 10% fetal bovine serum (Sigma, St. Louis, U.S.A.), 100 U/ml penicillin (Sigma) and 0.1 mg/ml streptomycin (Sigma). The cells were grown at 37°C in a 5% CO_2_ humidified incubator. Compounds were purchased from the following companies: celastrol and curcumin (Cayman, Michigan, U.S.A.), L-sulforaphane and resveratrol (Sigma), and betulinic acid (Tocris, Bristol, U.K.). Human recombinant TNFα was purchased from Sigma.

### Lentiviral vectors construction

To create the pRRL-PGKCBG99, the CBG99 luciferase gene was excised with NheI and XbaI from pGL3-CBG99 (Promega, Leiden, The Netherlands) and cloned in the MCS of pRRL-PGK (a kind gift from Prof. R. Hoeben [33]).

The bicistronic pLM-NF-κB PpyRE9 vector was constructed by amplifying the PpyRE9 luciferase gene from the vector pGex-6p-2-PpyRE9 (provided by Prof. B. Branchini [34]) using the following primers: forward primer 5′-GGCGGCCATGGAAGACGCCAAAAACATAAAG-3′ with a NcoI restriction site and reverse primer 5′-GTCTAGATTAGATTTTCCGCCCTTCTTGGCCTT-3′ with XbaI restriction site. Next, the PpyRE9 luciferase gene was cloned by replacing the luc gene in the pGL3 control vector (Promega) to create the pGL3-PpyRE9 vector. NF-κB promoter responsive elements were excised with KpnI and NcoI from the vector pGL4.32 [*luc2P*/NF-κB-RE/Hygro] (Promega) and cloned into the pGL3-PpyRE9 vector in order to create the pGL3-NF-κB PpyRE9 vector. Subsequently, the NF-κB PpyRE9 cassette was excised with KpnI and XbaI restriction enzymes, blunted and cloned into the bicistronic bidirectional pNFAT vector (kindly provided by Prof. M.R. Van de Brink [35]) in which the NFAT-CBRed cassette had been deleted. The resulting bicistronic lentiviral vector named pLM-NF-κB PpyRE9 was checked for orientation. The new plasmid contains the original Gaussia Luciferase [extGLuc] under the control of PGK promoter in one direction and the NF-κB PpyRE9 cassette in the other direction.

### Cell Transduction and selection

Self-inactivating lentiviruses were produced as previously described [33]. MDA-MB-231 cells were transduced in sequential steps. First, MDA-MB-231 cells were transduced with a lentivirus expressing pRRL-PGKCBG99 and selected by limiting dilution methods. Then, the obtained CBG99 luciferase positive cell line was transduced with the pLM-NF-κB PpyRE9 lentivirus and FACS-sorted using a polyclonal anti-Gaussia luciferase antibody (Nanolight tech., Pinetop, AZ).

### MTS and Luciferase assay

Triple colored MDA-MB-231 cells were seeded in 96-well plates and, 24 hours later, treated with different concentrations of puromycin (0.1 µg/ml – 10 µg/ml), a known selective antibiotic toxic to eukaryotic cells. After another 24 hours of incubation, plates were divided for the different analyses. An MTS colorimetric assay for assessing cell viability, CellTiter 96® AQ_ueous_ One Solution Cell Proliferation Assay (Promega, Leiden, The Netherlands) was used according to manufacturers' description. In brief, MTS solution was added to the medium for 2 hours after which the absorbance was measured at 490 nm with an ELISA microplate reader (VersaMax Molecular Devices, Sunnyvale, CA). To measure the luciferase activity biochemically, cells were washed with PBS, harvested in reporter lysis buffer and assayed for luciferase activity with a luminometer (SpectraMax L luminescence microplate reader, Molecular Devices). The cell viability curves were made for both MTS -and luciferase assays.

### Reporter gene activity assay

Triple colored MDA-MB-231 cells were seeded in 96-well dark plates (Greiner bio-one, Germany) at a concentration of 5000 cells/well, 24 hours prior treatment. The cell line was first validated for NF-κB responses by adding TNFα (10 ng/ml) for different time points of incubation (ranging from 2 to 72 hours). After establishing the optimal period of TNFα stimulation, the effect of the plant-derived compounds was investigated on TNFα-induced NF-κB activation in the MDA-MB-231 cells. For that, cells were treated with medium (negative control), TNFα (10 ng/ml, positive control), TNFα together with chemopreventive natural compounds: celastrol (0.1 µM–2.5 µM), sulforaphane (5 µM–100 µM), curcumin (10 µM–20 µM), resveratrol (1 µM–200 µM) or betulinic acid (0.1 µM–30 µM), followed by another 24 hours of incubation. Luciferase activity was determined as follows: the medium in plate was replaced with phosphate-buffered saline and D-Luciferin (Synchem, Germany) was added at a final concentration of 0.5 mM. Luciferase activity was measured using an IVIS Spectrum (Caliper, Alameda, CA, USA). The instrument stage was kept at 37°C and imaging set up was FOV C. Light output was measured using an open filter and a series of band pass filter (20 nm) ranging from 500 nm to 700 nm each for 5 sec, 5 min after substrate addition to live cells. All the data are expressed in photon-flux and analyzed with Living Image Software 4.0 (Caliper, Alameda, CA, USA). A spectral unmixing algorithm was applied to the images to separate the red and green luciferases as described previously [11]. Data were calculated depicting multiple region of interests (ROIs) corresponding to the well areas of the 96-well black plates in the images corresponding to the unmixing results. CBG luciferase activity in the green spectrum (peak emission wavelength 540 nm) has been used as an indication of cell vitality and the PpyRE9 luciferase activity in the red spectrum (peak emission wavelength 620 nm) as an indication of promoter activity. Measurement of extGLuc expression was carried out using native coelenterazine (Nanolight tech.) at a final concentration of 20 µM.

### Western blot analysis of P65 in nuclear extracts

To confirm the results of the reporter gene assays, western blot analysis was performed to test NF-κB activity in the nuclear extracts. MDA-MB-231 cells (1×10^6^ cells/well) were seeded in 6-well plates for 24 hours, then treated with medium (negative control), TNFα (10 ng/ml positive control), or TNFα (10 ng/ml) with celastrol (0.1 µM, 0.5 µM and 1 µM) or betulinic acid (0.5 µM, 1 µM, 10 µM and 30 µM). After 24 hours of treatment cells were collected, and nuclear extracts were made using Nuclear and Cytoplasmic extraction reagents (Thermo Scientific, Rockford, U.S.A.). Total amount of protein of each sample was determined by a Pierce BCA protein assay kit (Thermo Scientific, Rockford, U.S.A.). 15 µg of nuclear extracts was applied to a 10% SDS-PAGE and transferred onto a nitrocellulose membrane. After washing, the membrane was incubated with an anti-P65 polyclonal antibody in TPBS 1∶1000 dilutions (Cell Signaling Technology, Danvers, U.S.A.) overnight at room temperature. A GAPDH antibody (Cell Signaling Technology) was used to correct for the amount of total protein. The blots were washed, exposed to an HRP-conjugated secondary antibody for 1 hour, and finally detected using enhanced chemiluminescence (ECL) reagents (Thermo Scientific). Detection of ECL signals was performed with the IVIS Spectrum and quantification of bands using Living Image Software 4.0.

### Caspase 3/7 activity apoptosis assay

Triple colored MDA-MB-231 cells were seeded in a 96-well dark plate (Greiner bio-one) for 24 hours to attach. The cells were then treated with medium (negative control), or chemopreventive natural compounds, followed by another 4 hours or 24 hours of incubation. Rows of cells were then divided to estimate the effect of the different compounds on cell viability and apoptosis. Cell viability was established by the addition of D-luciferin to live cells; caspase 3/7 activity was determined by adding the luciferase pro-substrate, Z-DEVD-aminoluciferin using a Caspase-Glo 3/7 Assay (Promega). Upon activation of caspase-3 or -7, the DEVD peptide is cleaved off, releasing the aminoluciferin to react with luciferase generating measurable light [32]. The bioluminescent signals generated by the addition of the Z-DEVD-aminoluciferin were divided by the signals generated by the CBG99 luciferase upon addition of D-luciferin in order to correct the data set for the amount of viable cells. Data are reported showing the corrected signal for controls (only medium added) and the different treatments (medium containing the different plant-derived natural compounds).

### Triple colored MDA-MB-231 xenograft model

Triple colored MDA-MB-231 cells (2×10^6^) were implanted in the mammary fat pads of female athymic (BALB/c nu/nu) mice. D-luciferin (150 mg/kg) was injected intraperitoneally and imaging started 5 minutes later using a FOV C and a 30 sec acquisition time for both open filter and band pass filters acquisition. Mice were imaged weekly. Tumors reached a palpable size (around 150 mm^3^) after two to three weeks.

### Induction of NF-κB

When tumor growth reached an exponential increase in CBG-luc signals, mice were randomized in three groups. A control group received an intratumoral injection of PBS and an intravenous injection of PBS. TNFα (20 µg/kg) was injected intravenously in the second and third group. Next, these mice were given an intratumoral injection of celastrol (2 mg/kg) or PBS. Mice were imaged after the injection and after 24 hours with the same settings. Evaluation of NF-κB induction was performed by comparison of images before and 24 hours after treatment.

### Induction of apoptosis

To measure induction of apoptosis, triple colored MDA-MB-231 tumors were grown as described above, intratumorally injected with celastrol and after 4 and 24 hours Z-DEVD-aminoluciferin (50 mg/kg) was injected intraperitoneally. Activation of caspase 3/7 was measured 20 min after injection of substrate using an open filter and an exposure time of 30 sec.

### Immunohistochemistry

Tumors were surgically removed, fixed in 4% formaldehyde and further processed for paraffin embedment. Sections (6–7 µm) were made using a standard microtome and placed on glass slides. A polyclonal rabbit anti-Gaussia luciferase antibody (Nanolight tech.) and an anti-rabbit–FITC antibody were used to reveal the presence of Gaussia luciferase positive tumor cells.

### Statistical analysis

Each *in vitro* experiment was performed three times with six replicate samples per data point. A Student's t-test has been applied to determine statistically significant differences in the promoter activity between positive controls and treated conditions. For *in vivo* experiments wherein more than two groups were compared, one-way ANOVA followed by Tukey's post-hoc test was used to determine significant differences among treated groups.

## Results

### Generation of bioluminescent lentiviral vectors

**Figure 1:**
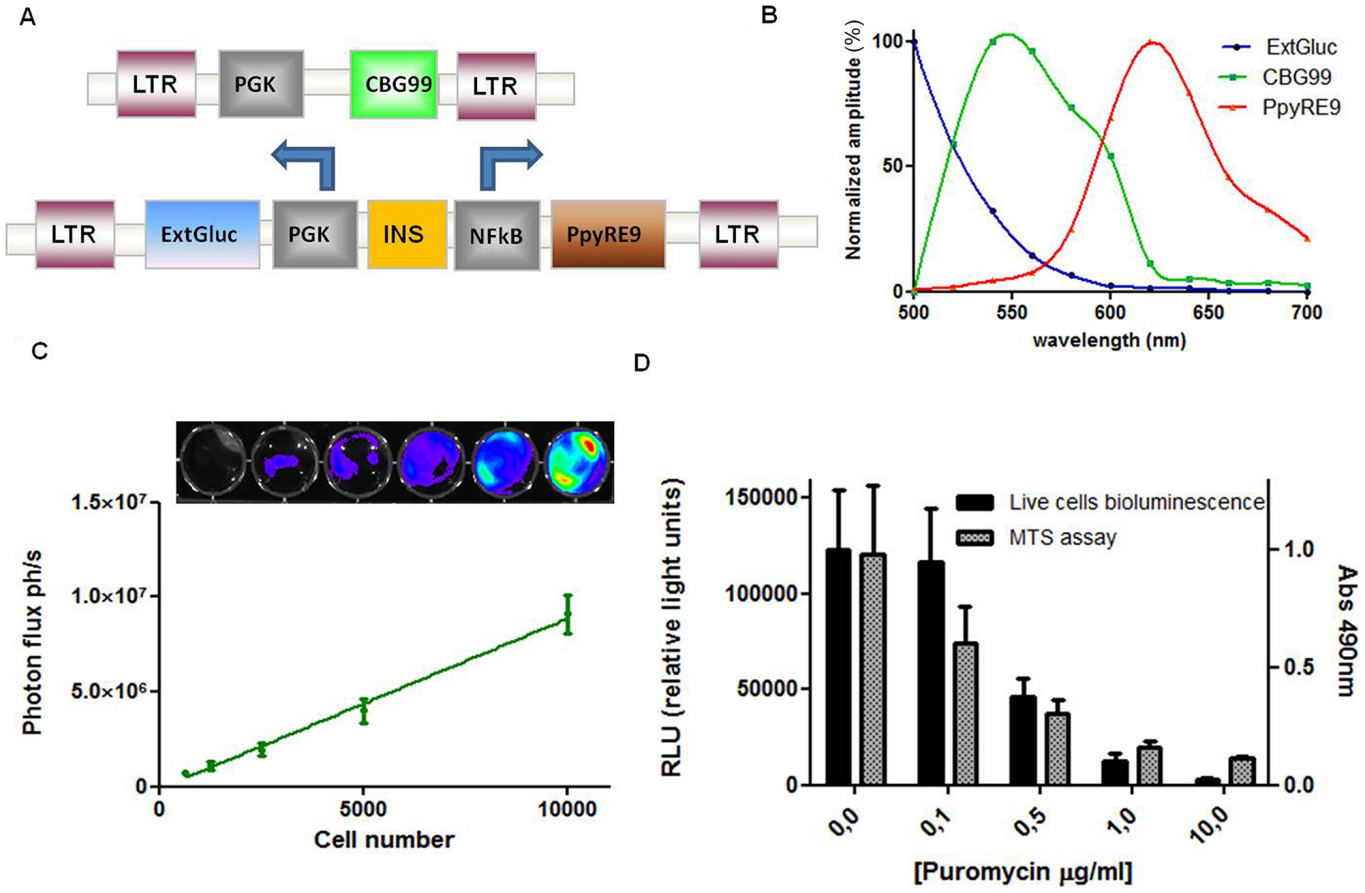
*In vitro* characterization of the triple color MDA-MB-231 cell line. (1.A) Schematic representation of the lentiviral constructs used for developing triple color MDA-MB-231 cells. (1.B) Graph representing the emission spectrum of the three luciferases used in the study. ExtGluc in blue, CBG99 in green and PpyRE9 in red. (1.C) Graph representing the correlation between the CBG99 luciferase signal and cells number in the MDA-MB-231 cell line. (1.D) Graph represents the correspondence between bioluminescent signal from CBG99 luciferase expressing MDA-MB-231 and the absorbance signals of MTS assay generating a kill curve using puromycin.

The pRRL-PGKCBG99 vector contained the CBG99 luciferase gene driven by a PGK promoter with a peak of emission at 540 nm after addition of D-luciferin substrate. The bicistronic vector pLM-NF-κB PpyRE9 consisted of a NF-κB PpyRE9 and a PGK-extGLuc cassette separated by an insulator sequence to ensure high induction of the promoters after integration of the vector (Figure 1.A). PpyRE9 luciferase has a peak of emission at 620 nm using D-luciferin as a substrate. The presence of the transmembrane Gluc reporter gene under the control of the constitutive promoter in the bicistronic vector represents the “third color” since the peak of emission of Gluc luciferase is 480 nm and needs the addition of a different substrate (e.g. coelenterazine) (Figure 1.B).

### The triple colored MDA-MB-231 cell line as a tool for monitoring vitality and NF-κB promoter activity

To create the triple colored MDA-MB-231 cell line, cells were first transduced with a lentivirus expressing pRRL-PGKCBG99 and selected by limiting dilution to obtain a population of positive cells in which the amount of light generated by the bioluminescent reaction strictly correlated with the cell number (R^2^ = 0.99) (Figure 1.C). The selected population underwent a second transduction with a lentivirus containing pLM- NF-κB PpyRE9. The transmembrane Gluc enabled a second round of sorting using an anti-Gluc antibody for indirect FACS-sorting. This resulted in a population of cells with high expression of the bicistronic vector.

An MTS and a luciferase assay were performed in parallel in a 96-well plate to demonstrate the correlation between cell death and luminescence. Firefly and click beetle luciferases are ATP dependent and therefore dying cells do not generate light. The protein synthesis inhibitor puromycin is toxic to eukaryotic cells and was used as a validating compound. As shown in Figure 1.D, the signal output reflected the puromycin (concentration range 0–10 µg/ml) effect on cells. In both assays the signal output reveals the killing of the cells due to antibiotic addition, demonstrating that the CBG99 green luciferase signal (emission peak: 540 nm) can be considered as an indication of vitality of cells.

**Figure 2:**
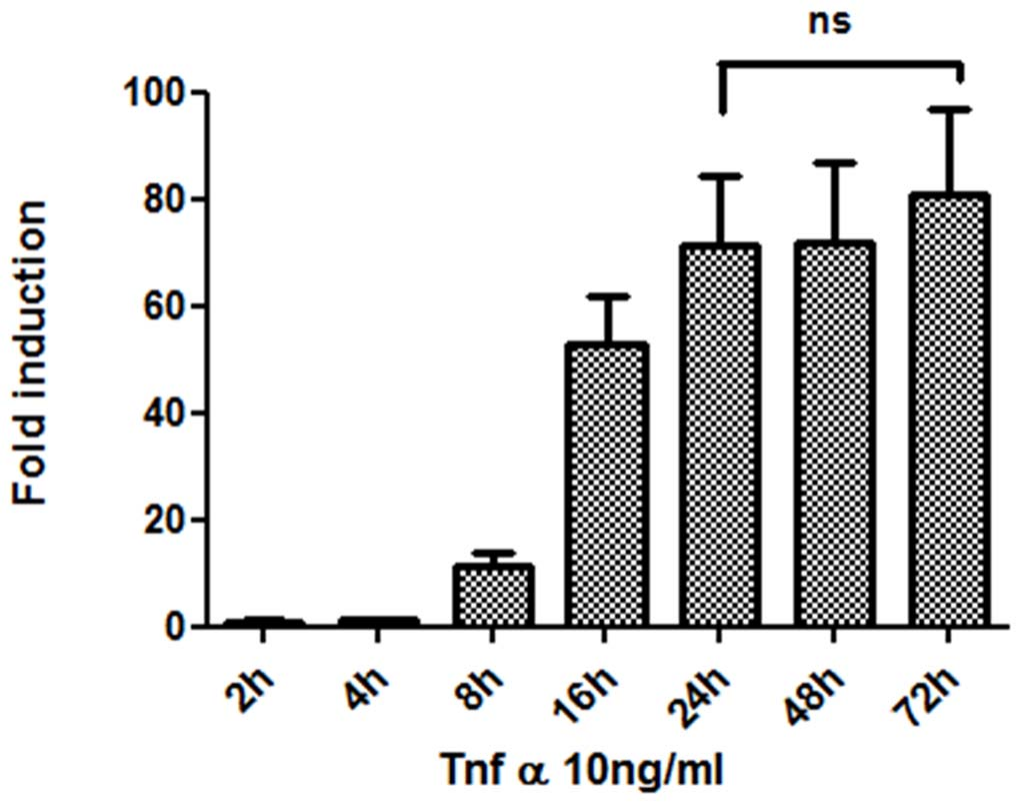
Time course analysis of TNFα-induced NF-κB activation in the triple color MDA-MB-231 cell line. Non-significant differences were detected in NF-κB activation between 24, 48 and 72 hours of stimulation with TNFα (10 ng/ml).

To further validate the cell line for NF-κB responses, we performed a time dependent analysis of TNFα- induced NF-κB activation. At a TNFα concentration of 10 ng/ml, 24 hours of incubation showed to be the time point at which a high NF-κB induction was achieved (Figure 2) with an average fold of induction of 70±13. No significant differences in NF-κB fold induction were detected when incubating TNFα for longer periods of time (48 and 72 hours).

### Effects of chemopreventive natural compounds on NF-κB promoter activity

**Figure 3:**
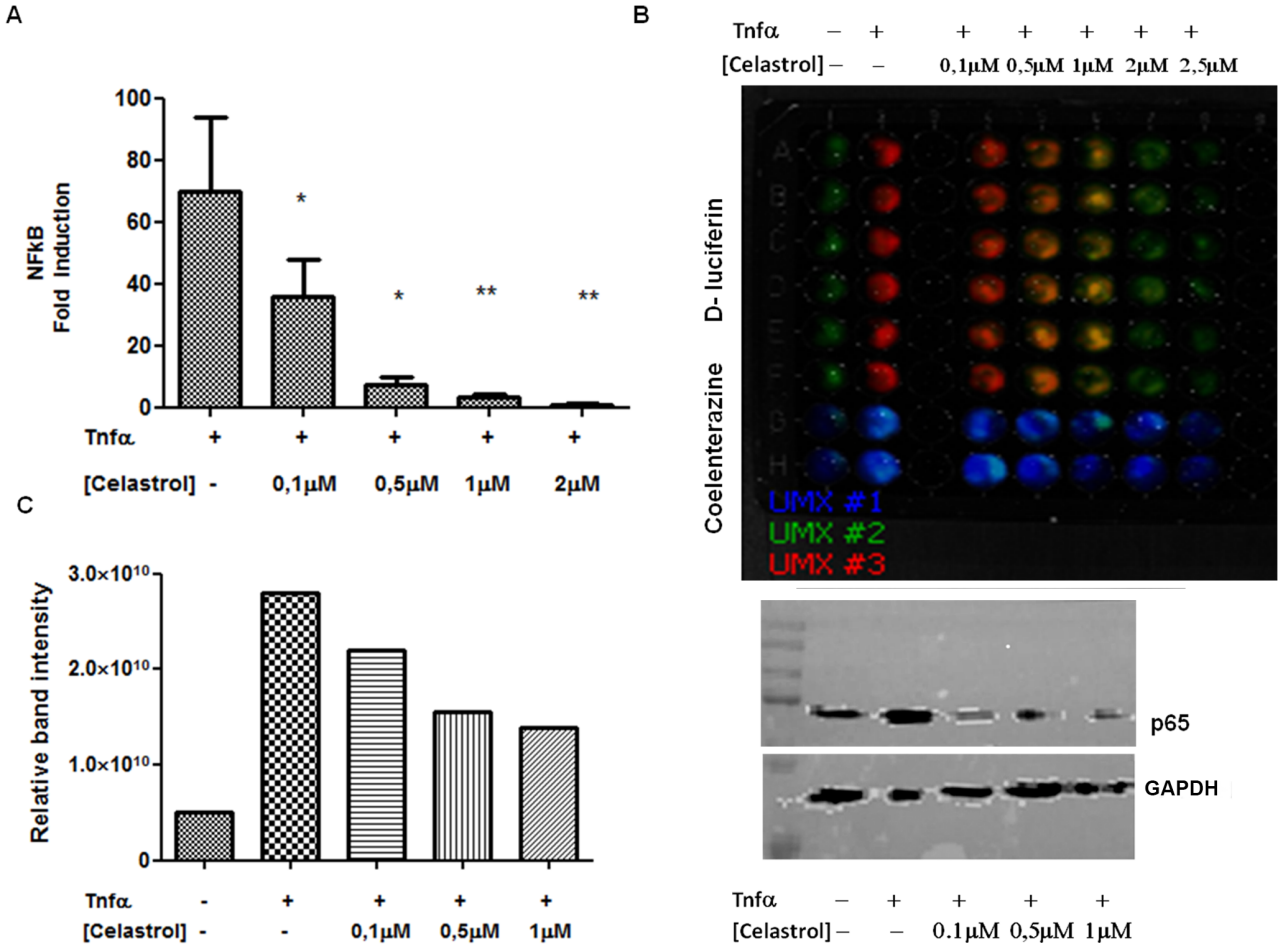
Celastrol has an inhibitory effect on TNFα-induced NF-κB signaling in MDA-MB-231 cells. (3.A) Graph representing the decrease in fold NF-κB induction in MDA-MB-231 cells using celastrol by the multicolor assay (* p value <0.05; ** p value <0.01). (3.B) Composite image of the unmixed spectrum of luciferases: green signals represent CBG99 emission, red signals represent PpyRE9 emission spectrum and blue signals represent ExtGluc expression in control cells (first column) and cells treated with TNFα in combination with increasing concentrations of celastrol (0.1–3 µM). (3.C) Left: graph reporting the relative band intensity of p65 protein from the nuclear extract of control sample and samples treated with TNFα (10 ng/ml) or TNFα + celastrol (0.1 µM; 0.5 µM and 1 µM). Right: p65 protein detection in nuclear extracts and GAPDH used as control protein.

The triple color assay was used to investigate the effects of chemopreventive natural compounds on NF-κB signaling. Data have been corrected for the number of cells (CBG99-green vitality signals) to normalize the fold of induction considering that some compounds induce cell death through apoptosis already after a short incubation time. For instance, already 4 hours of incubation with celastrol, an active ingredient of the traditional Chinese medicinal plant *Tripterygium wilfordii,* has been demonstrated to modulate the anticancer effect and induced apoptosis in a range of different cancer cells [36], [37]. Our results show that treatment of cells with celastrol clearly demonstrated a significant inhibition of TNFα-induced NF-κB promoter activity after 24 hours in a concentration range of 0.1–2 µM (p<0.05 for 0.1–0.5 µM; p<0.01 for 1–2 µM). Data were corrected for cell vitality as measured by the expression of CBG99 green luciferase (Figure 3.A). Figure 3.B shows a representative image of the assay performed after the application of the unmixing algorithm to the image: the green columns of wells show the detection of CBG99 green luciferase signals while the red-orange to yellow columns show the cells treated with TNFα plus increasing concentrations of celastrol. The decrease of the red signal is indicative for the inhibition of NF-κB promoter activity. The blue columns show the detection of Gaussia luciferase expressed after the addition of native coelenterazine and was used as an additional control for expression of the constructs. The intra-assay variability, calculated as the coefficient of variation (CV) of the green unmixed signal of the control wells, was around 6.0% when the assay was performed with six replicates. The inter-assay variability, calculated from nine consecutive cell passages, was much higher than the intra-assay variability with a pooled CV for all assay controls of 34%.

To confirm the effect of celastrol on TNFα-induced NF-κB signaling in MDA-MB-231 cells, western blot analysis of p65 protein in nuclear cell extracts was performed. As shown in Figure 3.C, TNFα treatment increased the amount of p65 in the nucleus while addition of celastrol had an inhibitory effect on the TNFα-induced NF-κB signaling. Data in the graph are normalized using GAPDH protein as a control for total protein load.

Resveratrol, a polyphenol found in high concentrations and thought to impart some of the beneficial properties of red wine [38], showed a significant inhibitory effect on TNFα-induced NF-κB promoter activity on MDA-MB-231 cells at concentrations ranging from 1 to 200 µM after 24 hours (p<0.05 for 1–10 µM; p<0.01 for 50–200 µM). Sulphoraphane, a potent chemopreventive and anti-inflammatory compound [39], [40], also exerted an inhibitory effect of TNFα-induced NF-κB promoter activity on MDA-MB-231 cells in a range of 5–100 µM with higher significance (p<0.01 from 20–100 µM). Also curcumin, derived from Turmeric (*Curcuma longa*) [41], that has been known for its anti-inflammatory properties, showed a similar effect already at 20 µM (p<0.05 for 10 µM; p<0.01 for 20 µM).

**Figure 4:**
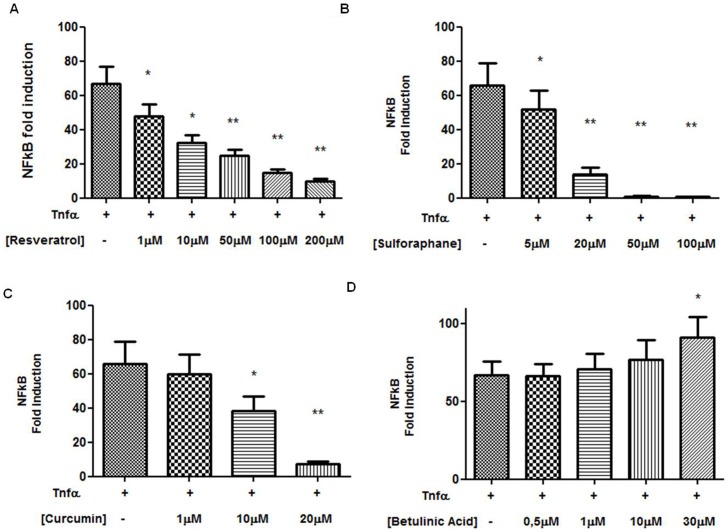
Effects of chemopreventive natural compounds on NF-κB promoter activity. Graphs representing the effect on NF-κB induction in MDA-MB-231 cells treated with different concentration of resveratrol (4.A), sulphoraphane (4.B), curcumin (4.C) and betulinic acid (4.D) (* p value <0.05; ** p value <0.01).

On the contrary, betulinic acid, a naturally occurring pentacyclic triterpenoid shown to exhibit a variety of biological activities, increased the TNFα-induced NF-κB promoter activity significantly at 30 µM demonstrating an additive effect in combination with TNFα activity after 24 hours [42]. The results are shown in Figure 4. In addition, western blot analysis confirmed the increased presence of p65 in the nuclear fraction of cells treated with TNFα and 30 µM of Betulinic acid (Figure S1).

### In vivo monitoring of NF-κB signaling by bioluminescence imaging

**Figure 5:**
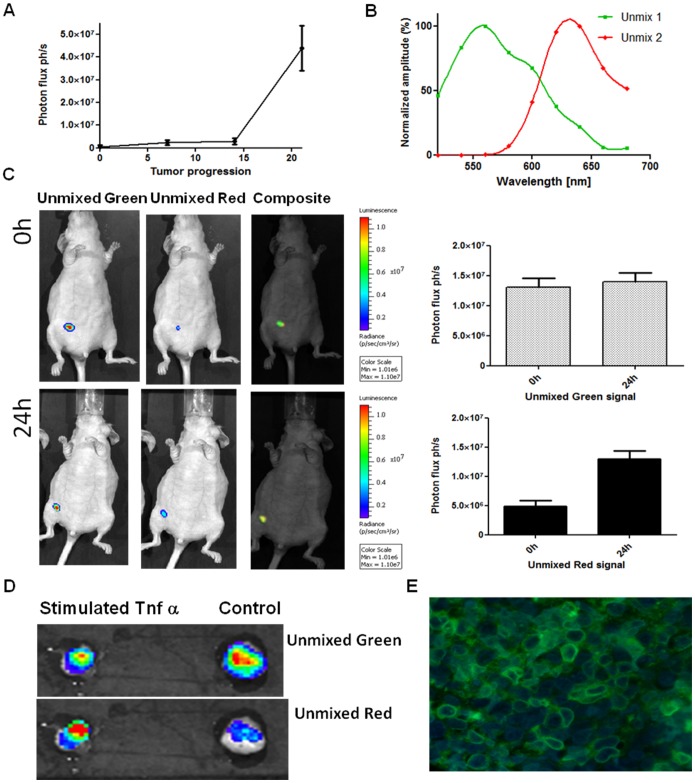
*In vivo* monitoring of NF-κB signaling by bioluminescence imaging. Growth curve of triple colored MDA-MB-231 cells implanted in the mammary fat pad of female athymic mice and measured by BLI (5.A). Red and green luciferase emission spectra as resulted from the spectral unmixing analysis applied to the series of acquired images (5.B). Representative picture of unmixed and composite images obtained with unmixing algorithm application at 0 and 24 hours after TNFα injection in a mouse model of breast cancer (5.C). On the left, the images corresponding to the green signal (vitality) while in the middle the images corresponding to the red signal (NF-κB induction). The graphs represent the average unmixed red and green signals obtained 0 and 24 hours after TNFα injection in three different mice. (5.D) Representative picture of unmixed images obtained with *ex vivo* analysis of tumors derived from mice challenged with or without TNFα. (5.E) Image showing Gluc expression on the membrane of cells derived from excised tumors and detected using a polyclonal anti-Gluc antibody and a FITC-conjugated secondary antibody.

Injection of the triple colored MDA-MB-231 cell line in the mammary fat pad induced tumor growth in mice. Tumor development was followed by CBG99 BLI for three weeks (Figure 5.A). At that timepoint animals received a dose of TNFα (20 µg/kg) to induce NF-κB dependent PpyRE9 red luciferase expression to validate dual color imaging *in vivo*. For the analysis the red/green signals were spectrally resolved (Figure 5.B) from the series of images taken using the 20 nm band pass filters applying a spectral unmixing algorithm. Animal receiving TNFα showed an increased light output in the image collected using red filters clearly detectable after 24 hours compared to control images taken at initial time point (t = 0 h) (Figure 5.C). An average 2.6 fold induction of NF-κB was measured by analysing the red signal at t = 24 h. Moreover, *ex vivo* imaging of the tumor directly after the *in vivo* imaging session confirmed the induction of NF-κB Ppy-RE9 luciferase (Figure 5.D). Additional immunohistochemistry of the excised tumors confirmed the presence of Gaussia luciferase on the surface of the cells (Figure 5.E).

**Figure 6:**
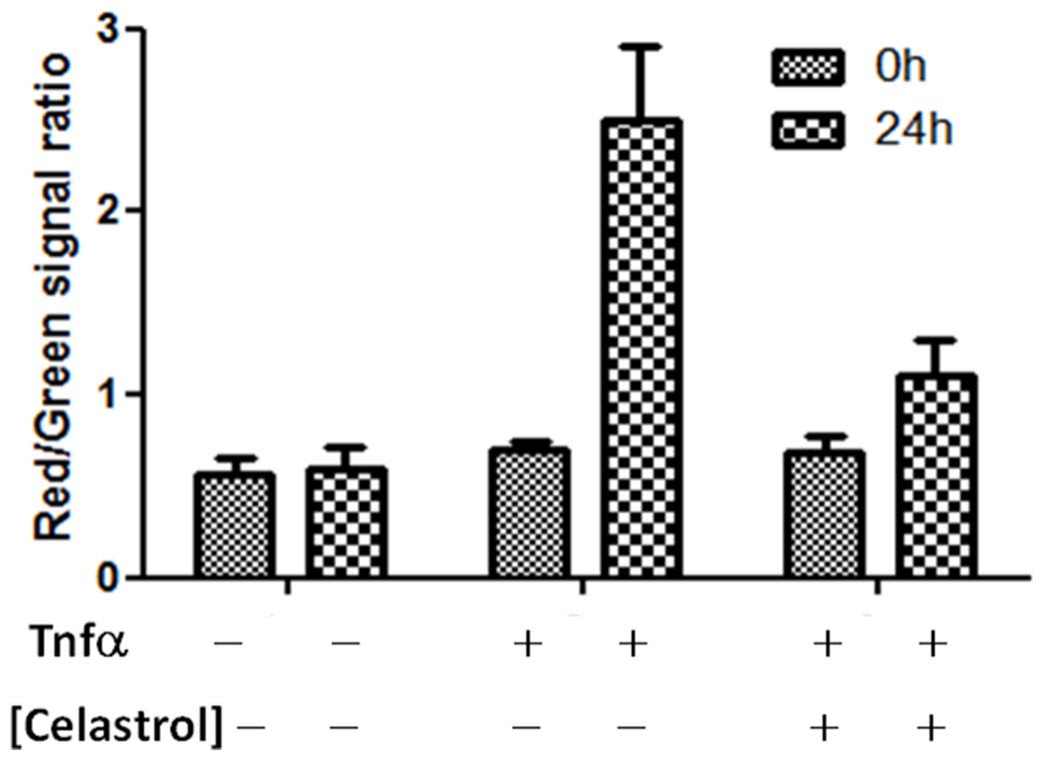
Celastrol has an inhibitory effect on TNFα-induced NF-κB signaling in breast cancer cells *in vivo*. Graph reporting the red/green signal ratio as measured in the group treated with TNFα or TNFα + celastrol (2 mg/kg) at 0 and 24 hours. Treatment with celastrol lowers the red/green signal ratio demonstrating to act on NF-κB signaling.

To confirm the efficacy of celastrol on NF-κB signaling *in vivo,* mice were injected with the triple colored cells and treated with TNFα (20 µg/kg) in combination with celastrol. Analysis of NF-κB induction, corrected for the green CBG99 signals at 0 and after 24 hours, revealed an increased red/green signal ratio (2.5±0.3) when treated with TNFα. This ratio is significantly different (p<0.05) from that of mice treated with celastrol (red/green signal ratio of 1.2±0.1) showing an *in vivo* inhibitory effect of celastrol on NF-κB activity (Figure 6).

### Caspase 3/7-mediated apoptosis after treatment with chemopreventive natural compounds

**Figure 7:**
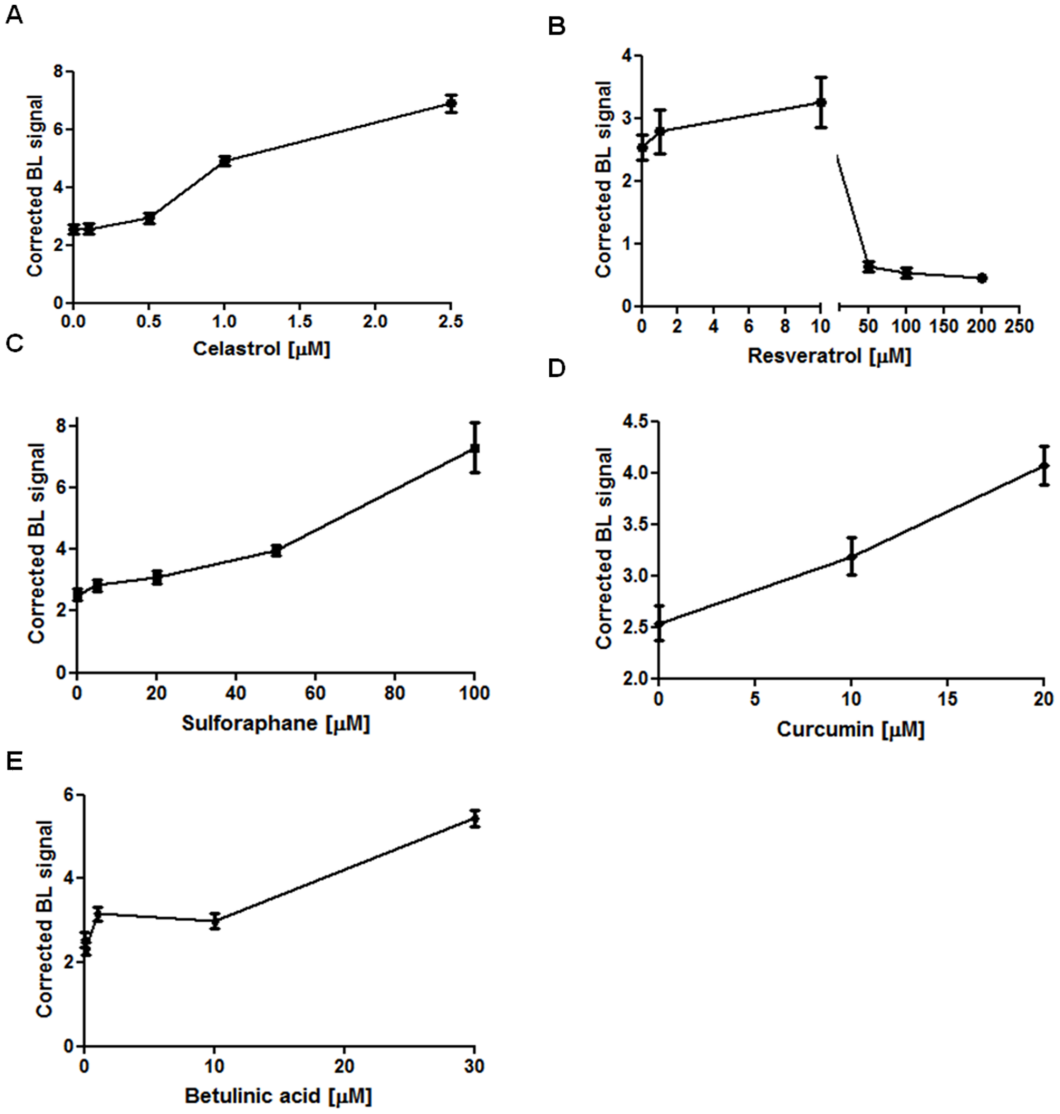
Effects of chemopreventive natural compounds on caspase 3/7-mediated apoptosis *in vitro*. Graph representing caspase 3/7 activity measured with the caspase 3/7 glo assay and corrected for cell vitality at 4 hours after treatment with celastrol (7.A) resveratrol (7.B), sulphoraphane (7.C), curcumin (7.D) and betulinic acid (7.E).

Induction of apoptosis by chemopreventive compounds was measured by using a firefly luciferase prosubstrate containing the DEVD tetrapeptide sequence. For these experiments, triple-colored MDA-MB-231 cells were incubated with the different natural compounds in the absence of TNFα. Constitutive CBG99 luciferase expression allowed to monitor the viability of cells in parallel with caspase 3/7 activity. After 4 hours of incubation celastrol, sulphoraphane, curcumin and betulinic acid induced high caspase 3/7 activity at 1 µM, 50 µM, 20 µM and 10 µM, respectively. Interestingly, resveratrol induced apoptosis at a concentration of 1 µM. However, caspase 3/7 activity was not significantly higher than basal induction of apoptosis control suggesting that resveratrol caused cell death most likely in a caspase 3/7 independent manner (Figure 7).

**Figure 8:**
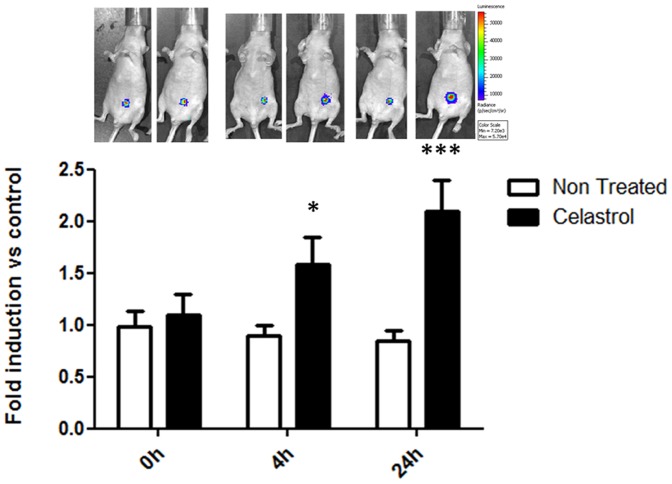
Celastrol induces caspase 3/7-mediated apoptosis *in vivo.* Graph reporting the *in vivo* caspase 3/7 activity measured using DEVD-luciferin in control and treated groups at 0 hours, 4 hours and 24 hours. A single treatment of celastrol (2 mg/kg) significantly increased the fold induction of caspase 3/7 activity at 24 hours compared to controls (* p value <0.05; ** p value <0.01).

Moreover, celastrol was also able to induce caspase 3/7 activity *in vivo* (Figure 8). After treatment with celastrol, mice showed significantly higher luminescence signals (fold of induction 1.5±0.2 p<0.05 at 4 hours and 2.1±0.3 p<0.001 at 24 hours) then basal luminescence signals at t = 0 hours. No significant differences in luminescence signals were found in non-treated control mice.

## Discussion

Induction of NF-κB signaling and apoptosis are some of the key processes in cancer. Plant-derived natural compounds have shown to affect these key processes and their use is emerging in cancer treatment. For this reason we generated a multicolor bioluminescence imaging platform to investigate the effect of plant-derived natural compounds on NF-κB activity and apoptosis for both *in vitro* and *in vivo* analyses.

We were able to reveal the effects of the plant-derived natural compounds (celastrol, resveratrol, curcumin and sulphoraphane) on NF-κB signaling and viability of the metastatic, triple negative human breast cancer cell line MDA-MB-231. Our results provide new useful insights into the effect of the different compounds on NF-κB transcriptional down regulation in human breast cancer cells and open new possibilities to investigate NF-κB signaling in other established human cancer cell lines by implementing the triple colored bioluminescent reporter cell system.

Lentiviral vector technology is an efficient method to stably and randomly integrate foreign DNA in dividing and non-dividing cells. The possibility of sequential transductions allowed us to select the cell population with the desired expression profile. Once the stable cell line is generated, the time of the assay is consistently reduced compared to traditional transcriptional assays because the transient transfection step is eliminated. Moreover, the off-target effects caused by the transfection reagents, i.e. cytotoxicity and impact on transcript level, are now avoided [43].

The choice of the PGK promoter for mammalian cancer cells ensured a good level of constitutive expression of the green CBG99 luciferase. The good correlation between the MTS assay and CBG99 signals confirmed that CBG99 could function as an indicator of cell viability. Consequently, the green emitting luciferase has been employed to measure cell viability in *in vitro* assays but also in *in vivo* using bioluminescence imaging.

Introduction of a bicistronic bidirectional vector in the CBG luc expressing cell line allowed the creation of a triple colored MDA-MB-231 cell line expressing the red PpyRE9 luciferase controlled by the NF-κB promoter and transmembrane form of Gaussia luciferase, driven by the PGK promoter. The validation of this triple colored MDA-MB-231 cell line revealed that the NF-κB induction by TNFα could easily be monitored already after 4 and 8 hours with a very strong induction after 24 hours. This time point was optimal when performing inhibition experiments with natural compounds. The 5 copies of the NF-κB response element located upstream of the minimal CMV promoter have proven to result in high NF-κB induction by TNFα [44].

Since celastrol has already been demonstrated to act on the NF-κB pathway [36] we validated our cell based assay by showing that the NF-κB inhibition coincided with a decreased p65 protein expression in nuclei of MDA-MB-231 cells. We also found that the dose that caused NF-κB inhibition also caused caspase-3/7 dependent apoptosis after 4 hours already at a concentration of 0.1 µM [37], [45].

In analogy we tested other natural compounds (e.g. resveratrol, curcumin, sulphoraphane and betulinic acid) to investigate their activity on TNFα-induced NF-κB promoter activity and whether they can induce caspase-3/7 dependent apoptosis. Results showed that resveratrol significantly inhibited the TNFα-induced NF-κB promoter activity already at a concentration of 1 µM and induced caspase-3/7 dependent apoptosis as early after 4 hours as described earlier [46]–[48]. At higher concentrations it was not possible to detect caspase 3/7 activity due to massive cell death caused by (a) caspase 3/7 independent mechanism(s).

Addition of sulphoraphane resulted in a reduction on TNFα-induced NF-κB promoter activity and an induction of caspase 3/7 dependent apoptosis which is in accordance with previous literature [39], [40]. Several studies revealed that sulphoraphane not only acts as a chemopreventive drug, but also as a chemotherapeutic agent in colon and pancreatic tumors [49] and recently its effect on breast cancer stem cells has been demonstrated [50]. Interestingly, its capacity to reactivate estrogen receptor (ER) expression in ER-negative breast cancer cells by active chromatin modifications has also been shown [51].

As previously demonstrated [41], [52], a significant effect of curcumin on the reduction of TNFα-induced NF-κB promoter activity and on caspase-3 activity was seen at a concentration of 20 µM. Curcumin has also been proven to be effective in anticancer therapy not only as a chemopreventive drug but also as a coadjuvant in breast cancer therapy. Curcumin and its more potent analogues have been shown to kill [52], [53] breast cancer cells and reduce metastatic potential. However, bioavailability of curcumin *in vivo* still represents a problem for its clinical use. More potent analogues of curcumin have been described that lower the effective dose *in vivo*. Recent results show that improved curcumin analogues like UBS109 and EF31 [54], are already effective at a dose of 1 µM, and would represent a great improvement towards the clinical use of curcumin in adjuvant therapy of breast cancer (Mezzanotte L, unpublished results). Formulations based on nanomaterials for effective biodistribution of curcumin have already been used in metastatic breast cancer [55]–[57]. Nanotechnology-based delivery systems for more potent curcumin analogues to improve their bioavailability would enhance the efficacy of cancer treatment.

Our *in vitro* results from experiments on apoptosis and NF-κB signaling reinforce the hypothesis that celastrol, and less potently resveratrol, can be used as adjuvant therapy for treatment of triple negative breast cancer since they showed inhibitory effects of NF-κB signaling and induction of apoptosis on the micromolar scale. Sulphoraphane and curcumin are also effective, although to a lesser extent. Betulinic acid has no inhibitory effect on NF-κB signaling although still effective for cancer treatment since it causes apoptosis of cancer cells that is probably not mediated by NF-κB downregulation. Considering the low coefficient of variation for both intra-assay and low inter-assay variability obtained in the 96-well plate format, it would be possible to adapt the triple colored MDA-MB-231 cell based assay to a high throughput screen platform by optimizing culture conditions and automation of procedures required for performing the screening [58]. Moreover, the same multicolor approach can be applied to monitor different pathway in other cells lines.

The relatively high average cell photon fluxes of the triple color MDA-MB-231 cell line *in vitro* (900 and 1500 ph/sec/cell for CBG99 and extGluc, respectively) make it possible to perform BLI microscopy on single cells. Preliminary data showed the feasibility of single cell imaging using bioluminescence microscopy by adding 0.2 mM D-luciferin or 20 mM of native coelenterazine to cultured cells (data not shown). For separation of emitted colors a bioluminescent microscope equipped with the right optical filters is needed [59]. Live cell microscopy imaging requires far less sample than traditional biochemical assays and adds a critical new dimension to the data allowing studying the responses of various activators or inhibitors at different concentrations at a cellular level over time.

The development of the triple color breast cancer cell line also demonstrated to be useful for *in vivo* studies improving the predictability of preclinical models for drug testing. After injection of cells in the mammary fat pad, tumor growth could be imaged by means of bioluminescence imaging. Tumor growth was followed for three weeks measuring the signals derived from CBG99 luciferase. At that point tumors reached a palpable size and mice were challenged with TNFα in order to test NF-κB promoter activity. After induction by TNFα, bioluminescent images were taken using a series of band pass filters and a spectral unmixing algorithm was applied to discriminate the CBG99 green signals from the PpyRE9 red signals [11]. Our results showed the possibility to apply multicolor image analysis and evaluate drug effects on the NF-κB signaling *in vivo* with the use of one single substrate injection when total light emission from tumor is higher than 10^7^ ph/s (at day 21). *Ex vivo* analysis of the tumors confirmed that mice challenged with TNFα showed an increased signal in the red region of the spectrum due to the induction of NF-κB -driven PpyRE9 expression. Additional immunohistochemistry showed the presence of Gaussia luciferase on membranes of cancer cells and revealed that the expression of the bicistronic construct is stably maintained in approximately 90% of the breast cancer cells during their growth *in vivo*. The major advantage of using extGluc in our model was the possibility to sort cells by FACS and to detect them both *in vitro* and *ex vivo*. Therefore, extGluc can be used as a specific tag that has no toxicity in cells in contrast to some fluorescent proteins [60], [61].

Animals were treated with TNFα to induce NF-κB -driven PpyRE9 expression alone or in combination with celastrol to determine the *in vivo* effect of a single dose of drug on NF-κB promoter activity. Celastrol was chosen since it was the most potent NF-κB inhibitor among the tested compounds and was injected intratumorally to ensure a good bioavailability. The ratio between the red unmixed signal and the green unmixed signal allowed measuring the effect of celastrol on NF-κB promoter activity. Additionally, we were able to image the caspase 3/7 activity in real time and determined the effect of celastrol after 24 hours in mice using DEVD-luciferin substrate as reported in previous work [32].

In conclusion, our developed multicolor optical imaging platform allowed the simultaneous investigation of NF-κB signaling and apoptosis induced by drugs as confirmed by both our *in vitro* and *in vivo* studies using celastrol. This new imaging platform opens up new opportunities to test the efficacy and bioavailability of (new formulations of) compounds that act upon NF-κB signaling for single time point experiments in small animals.

## Supporting Information

Figure S1 **Increased presence of p65 in the nuclear fraction of MDA-MB-231 cells after treatment with TNFα and Betulinic acid.** Western Blot analysis. Left: graph reporting the relative band intensity of p65 protein from the nuclear extracts of control sample and samples treated with TNFα (10 ng/ml) or TNFα + betulinic acid (0.5 µM; 1 µM; 10 µM and 30 µM). Right: p65 protein detection in nuclear extracts and GAPDH used as control protein.
(TIF)
