# Supplementary figures and images for: A New Multicolor Bioluminescence Imaging Platform to Investigate NF-κB Activity and Apoptosis in Human Breast Cancer Cells
Source: PLoS One. 2014 Jan 17;9(1):e85550. doi: 10.1371/journal.pone.0085550 (PMC3894999; doi:10.1371/journal.pone.0085550)

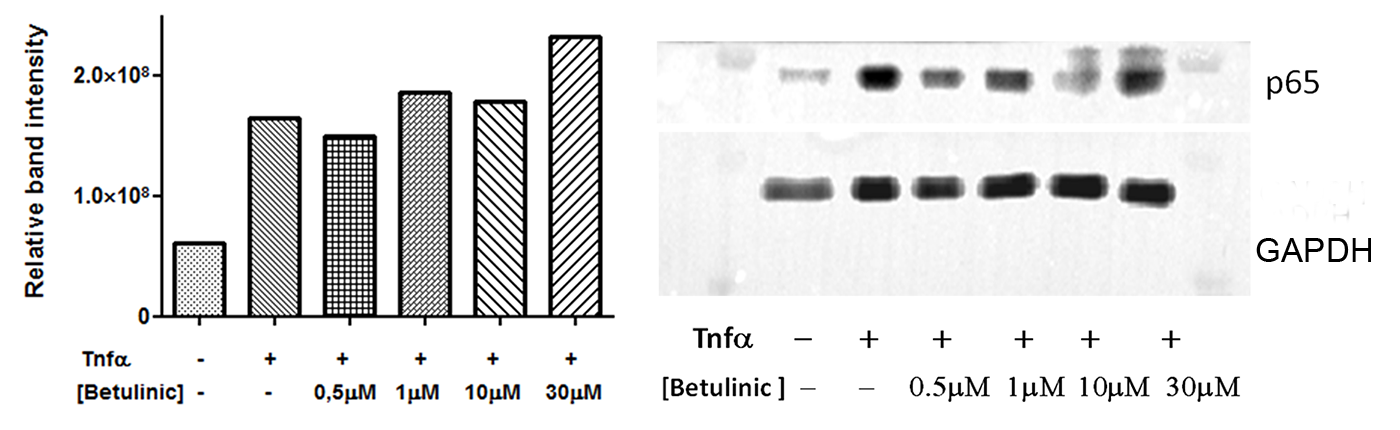

Supplement: Figure S1 — Increased presence of p65 in the nuclear fraction of MDA-MB-231 cells after treatment with TNFα and Betulinic acid. Western Blot analysis. Left: graph reporting the relative band intensity of p65 protein from the nuclear extracts of control sample and samples treated with TNFα (10 ng/ml) or TNFα + betulinic acid (0.5 µM; 1 µM; 10 µM and 30 µM). Right: p65 protein detection in nuclear extracts and GAPDH used as control protein. (TIF) [file pone.0085550.s001.tif]
